# Effect of Supplementing Seaweed Extracts to Pigs until d35 Post-Weaning on Performance and Aspects of Intestinal Health

**DOI:** 10.3390/md19040183

**Published:** 2021-03-26

**Authors:** Stafford Vigors, John O’Doherty, Ruth Rattigan, Torres Sweeney

**Affiliations:** 1 School of Agriculture and Food Science, University College Dublin, Belfield, Dublin 4, Ireland; staffordvigors1@ucd.ie (S.V.); ruth.rattigan@ucdconnect.ie (R.R.); 2 School of Veterinary Medicine, University College Dublin, Belfield, Dublin 4, Ireland; torres.sweeney@ucd.ie

**Keywords:** laminarin, fucoidan, gastrointestinal tract, microbiome, transcriptome, swine, post-weaning, antibiotic alternatives

## Abstract

The objective of this study was to examine the effects of feeding laminarin (LAM) and fucoidan (FUC) enriched seaweed extracts up to d35 post-weaning on measures of animal performance, intestinal microbial and transcriptome profiles. 75 pigs were assigned to one of three groups: (1) basal diet; (2) basal diet + 250 ppm fucoidan; (3) basal diet + 300 ppm laminarin with 7 replicates per treatment group. Measures of performance were collected weekly and animals sacrificed on d35 post-weaning for the sampling of gastrointestinal tissue and digesta. Animal performance was similar between the basal group and the groups supplemented with FUC and LAM (*P* > 0.05). Pigs fed the basal diet had higher alpha diversity compared to both the LAM and FUC supplemented pigs (*P* < 0.05). Supplementation with LAM and FUC increased the production of butyric acid compared to basal fed pigs (*P* < 0.05). At genus level pigs fed the LAM supplemented diet had the greatest abundance of *Faecalbacterium*, *Roseburia* and the lowest *Campylobacter* of the three experimental treatments (*P* *<* 0.05). While neither extract had beneficial effects on animal performance, LAM supplementation had a positive influence on intestinal health through alterations in the gastrointestinal microbiome and increased butyrate production.

## 1. Introduction

The weaning process is a stressful event in the pig’s life, characterized by intestinal and immune dysfunctions that result in impaired feed intake, health and growth [1]. The post-weaning growth check is characterized by an increased proliferation of pathogenic bacteria, in particular β-hemolytic enterotoxigenic *E. coli* serotypes, causing post-weaning diarrhea [2]. Antibiotic growth promoters are an effective means to reduce pathogenic bacteria such as *E. coli* and enhance growth rates in the immediate post-weaning period. However, the use of antibiotics as growth promoters was banned in the EU in 2006 (EC regulation no. 1831/2003). Since the ban on antibiotics, pharmacological doses of zinc oxide (ZnO) are used to reduce the incidence of diarrhea and improve performance in the post-weaning period. However, mounting concerns over the accumulation of ZnO in soils and a link between ZnO usage and antibiotic resistance have meant a phasing out of its usage by 2022 (Regulation (EU) 2019/61 on Veterinary Medicines and Regulation (EU) 2019/4 on Medicated Feed). This legislation also adds further restrictions to the use of antibiotics for prophylaxis or metaphylaxis. Therefore, there has been a focus on finding more natural means to improve performance and digestive health in pigs during the post-weaning period.

This search for natural alternatives has focused on a wide variety of substances such as organic acids, yeast derivatives, glucans, mannans, herbs and spices amongst many others [3]. One alternative that has been explored is the use of seaweed extracts such as laminarin (LAM) and fucoidan (FUC) as an alternative to antibiotics and ZnO [4]. The extract LAM is a β-glucan composed of β-1-3-d-glucan with β-1-6 branch chains that vary with species in distribution and length, while FUC represents a class of fucose-enriched sulfated polysaccharides extracted from the extracellular matrix of brown algae, with l-fucose 4-sulfate as the major component [4].

The feeding of seaweed extracts, such as laminarin and fucoidan, in the immediate post-weaning period illicits multiple beneficial physiological adaptions in the gastrointestinal tract, that can improve pig performance during this challenging time. A number of studies have examined the effects of feeding laminarin and fucoidan for 14 days post-weaning [5,6,7]. In the study of Rattigan et al. [5], pigs were offered 3 levels of a laminarin rich extract to identify the most appropriate inclusion level. The 300 ppm laminarin supplemented group exhibited the greatest beneficial effects with improvements in animal performance with accompanying positive alterations in small intestinal morphology, microbial populations and gene expression profiles. Based on these positive findings, a more detailed analysis of the microbiome was conducted by Vigors et al. [7], where increased abundance of *Prevotella* and reductions in the family *Enterobacteriaceae* were reported in the laminarin supplemented group. In addition, while pigs supplemented with 250 ppm fucoidan did not see any improvement in performance, they had improved faecal scores and increased concentrations of total volatile fatty acids in the colon but had alterations in gene expression and microbial profiles which potentially could have negative connotations for animal health in the longer term [8]. Hence, in this experiment, the feeding of the experimental diets was continued past d14 until d35 to assess the longer-term effects on animal performance and intestinal health. This strategy will establish whether the feeding of LAM continues to be beneficial until d35 or beneficial effects are limited to the immediate post-weaning period. Therefore, the objective of this research was to examine the effects of feeding seaweed extracts (SWE) up to d35 post-weaning on animal performance with a focus on immune and microbial profiles in the gastrointestinal tract.

## 2. Results

### 2.1. Characterization of Seaweed Extracts

The LAM extract was obtained from *Laminaria Digitata* and contained 653.2 g of laminarin per kg DM, 190 g/kg fucoidan per kg DM, 5 g/kg phlorotannin per kg DM, 51 g/kg mannitol per kg DM, 40 g/kg alginates per kg DM (Table 1).

The FUC rich extract was obtained from *Ascophyllum nodosum* and contained 441 g of fucoidan per kg DM, 25.9 g laminarin/kg DM, 135g alginates/kg DM, 43.8 g mannitol/kg DM, 34.8 g phlorotannin’s/kg DM.

### 2.2. Animal Performance

The average body weights of the pigs on the SWE diets were the same as the control group at the end of the experimental period (*P* > 0.05; Table 2). While no significant effects of dietary supplementation with either LAM or FUC were identified for measures of ADFI or ADG compared to the control group (*P* > 0.05), pigs fed the FUC supplemented diet had lower G:F ratio compared to the control (*P* < 0.05; Table 2).

### 2.3. Volatile Fatty Acids

Pigs on the LAM diet had the highest concentrations of total volatile fatty acid (VFA) and butyrate compared to the FUC supplemented group and the basal group (*P* < 0.05; Table 3).

### 2.4. Differential Abundance Analysis of Bacterial Taxa in Colonic Digesta and Alpha Diversity Analysis

Next-generation sequencing of the 16S rRNA gene using the Illumina Miseq platform was conducted to examine the impact of feeding the experimental diets on the intestinal microbiome. This analysis assessed the impact on measures of diversity (Table 4) and differential abundance analysis that was conducted at the taxonomic levels of Phylum, Family and Genus. The analysis of all taxa is presented in the Supplementary Tables (S1–S3) while significant data are presented in Table 5.

Pigs fed the basal diet had the highest alpha diversity of the three experimental groups based on Observed, Chao1, ACE, Shannon and Fisher measures of alpha diversity (*P* < 0.05). The LAM and FUC supplemented pigs had similar levels of alpha diversity.

**Phylum:** Pigs on the LAM supplemented diets had increased abundance of Firmicutes and Spirochaetes compared to pigs fed the basal and FUC supplemented diets (*P* < 0.05). Pigs fed the FUC supplemented diet had the highest abundance of Proteobacteria compared to both the basal and LAM diets (*P* < 0.05) with pigs fed a LAM supplemented diet having lower abundance of Proteobacteria compared to the basal diet (*P* < 0.05).

**Family:** Pigs on the LAM supplemented diets had lower *Campylobacteraceae* and increased *Spirochaetaceae* compared to the other two groups (*P* < 0.05) and increased *Ruminococcaceae* compared to the FUC supplemented group (*P* < 0.05).

**Genus:** Pigs on the FUC supplemented diet had lower *Faecalbacterium* and higher *Campylobacter* compared to pigs on both the LAM and basal diets (*P* < 0.05). Pigs on the LAM supplemented diet had lower *Campylobacter* compared to the other two diets (*P* < 0.05). Pigs on the basal diet had increased *Alloprevotella* compared to pigs fed the SWE supplemented diets. LAM supplementation increased *Roseburia* compared to the pigs fed the control diet (*P* < 0.05), while also increasing *Treponema* compared to pigs fed both the basal and the pigs fed the diet supplemented with FUC (*P* < 0.05).

### 2.5. Differential Expression Analysis of Genes Related to Nutrient Absorption and the Intestinal Immune Response

The Nanostring nCounter technology was employed to examine the effect of LAM and FUC supplementation on the expression of genes related to intestinal health and functionality. The expression profile of 32 genes in the small intestine were measured, as described in the materials and methods. All gene expression data is presented in Supplementary Tables (S4–S6) while significant data is presented in Table 6.

#### 2.5.1. Differential Expression Analysis of Digestive Enzyme and Nutrient Transporter gene expression

Duodenum: FUC supplementation increased the expression of *SLC6A19* compared to LAM supplementation, while FUC supplementation increased the expression of *CNDP1* compared to both LAM supplementation and the basal group (*P* < 0.05). Pigs fed the basal diet had increased expression of *SLC16A10* compared to both LAM and FUC supplementation.

Jejunum: LAM supplementation increased the expression of *MCT1/SLC16A1* compared to FUC supplementation and the basal group (*P* < 0.05).

Ileum: FUC supplementation decreased the expression of *SLC2A2*, *GCG*, *FABP2*, *SLC5A1* and *SI* compared to the basal group (*P* < 0.05). Pigs fed the basal diet had increased expression of *CNDP1* compared to the FUC and LAM supplemented groups (*P* < 0.05).

#### 2.5.2. Differential Expression of Markers of Immune Response and Intestinal Integrity

Jejunum: Pigs on the FUC supplemented diet had increased expression of *CXCL8* compared to both LAM supplementation and the basal diet (*P* < 0.05). Pigs on the LAM supplemented diet had reduced *MUC1* compared to the basal and LAM supplemented diet (*P* < 0.05). Pigs on the basal diet had higher expression of *OCLN* compared to pigs fed the FUC and LAM supplemented diets (*P* < 0.05).

Ileum: pigs fed the FUC supplemented diet had increased expression of *TGFB1* and *TNF* compared to the control and LAM groups (*P* < 0.05). Supplementation with FUC decreased expression of *CLDN5*, *CLDN3* and *OCLN* compared to the basal diet (*P* < 0.05).

## 3. Discussion

While supplementation with LAM was previously shown to be effective in improving pig performance from weaning until day 14 post-weaning [5], the results of this study suggest that, under good sanitary conditions, there is no additional benefit on performance to continued supplementation with either LAM or FUC supplementation up to day 35 post-weaning. It is important to note, however, that pigs fed the LAM supplemented diet had increased colonic butyrate production and positive alterations in the colonic microbiome characterized by reduced *Campylobacter* and increases in the genus *Roseburia* and *Faecalbacterium*. Such effects could contribute to a better host immune response to any potential environmental/pathogen challenge during this time period.

There were substantial differences in the microbiome profile between the three experimental groups. There was a contrasting effect between the two SWE supplemented treatments, particularly in relation to changes in the phylum Proteobacteria. The pigs fed the LAM supplemented diet had a decrease in this phylum, while pigs fed the FUC supplemented diet had an increase in the abundance of this phyla compared to pigs fed the basal diet. These differences were supported at both family and genus taxonomic levels with differences in the family *Campylobacteraceae* and the genus *Campylobacter.* The magnitude of the differences was interesting as pigs fed LAM had 23 times lower *Campylobacter* compared to pigs fed the basal diet, while pigs fed the FUC supplemented diet had 4 times higher *Campylobacter* compared to pigs fed the control diet. *Campylobacter* is a major foodborne pathogen often solely linked to infections from broilers through *C. jejuni*, however, *C. coli* is implicated with infections originating from swine [9]. A potential anti-bacterial effect following the addition of LAM is potentially of major benefit to the pig industry [10]. While *Campylobacter* is often considered to form part of the commensal flora of pigs and cause no negative impact on animal performance, there is some debate as high counts of *Campylobacter* are often observed in conjunction with other bacteria during incidences of diarrhea [11]. From either the aspect of reducing a zoonotic pathogen or improving intestinal health, the observed effects on *Campylobacter* by the addition of LAM can be considered a positive finding while the large increase in *Campylobacter* following the addition of FUC is likely to have negative connotations.

Coinciding with the lowest abundance of Proteobacteria in this study, pigs fed the LAM supplemented diet had the greatest abundance of the phylum Firmicutes. This increase was predominantly related to increases in the *Ruminococcaceae* and the *Lachnospiraceae* families. At genus level this coincided with differences in *Faecalbacterium* and *Roseburia.* Within the genus *Faecalbacterium* only one species has been identified in the literature, which is *Faecalbacterium prausnitzi*, implying that this change at genus level relates to a change in abundance of this species. This genus has been associated with increased intestinal health based on a low incidence of post-weaning diarrhea in pigs [12]. In humans, *Faecalbacterium prausnitzi* is used as an indicator of intestinal health and is also considered to be a very promising probiotic for human use [13,14]. The end products of carbohydrate fermentation by *Faecalbacterium prausnitzi* are formate, lactate and substantial quantities of butyrate [15]. Further corroborating the potential improved gut health in pigs fed LAM are an increase in the genus *Roseburia*. *Roseburia* is a member of the Firmicutes phylum and is associated with the production of butyrate [8]. Therefore, the increase in *Faecalbacterium and Roseburia* could be associated with the increase in butyric acid identified in pigs from this study fed the LAM supplemented diet. Butyrate is produced from bacterial fermentation to provide energy to colonocytes. It also has functions as a cellular mediator, regulating multiple functions of gut cells and beyond, including gene expression, cell differentiation, gut tissue development, immune modulation, oxidative stress reduction and diarrhea control [16]. These changes suggest an improvement in gut health in pigs fed the LAM supplemented diet.

In contrast to the clear beneficial effects of supplementation with LAM on members of the Firmicutes and Proteobacteria phyla, the increase in abundance of the Spirochaetes phylum are less clear. Within this phylum associated changes were identified with increases in the family *Spirochaetaceae* and the genus *Treponema* were also identified, with pigs fed the LAM supplemented diet having greater abundance than both the two other experimental groups. The genus contains both pathogenic as well as commensal bacteria [17]. Pathogenic bacteria include *Treponema pallidum*, and others such as *T. denticola* (Tde), *T. putidum* (Tpu) *T. pedis* (Tpe), *T. brennaborense* (Tbr), and *T. paraluiscuniculi* (Tpar) which are the causative agents of various disease in both humans and animals [18]. Porcine intestinal Spirochaetes form a diverse group of organisms including the beta-hemolytic *Serpulina*/*Treponema hyodysenteriae* which is the main etiological agent of swine dysentery [19]. While the classification of OTUs at species level is unreliable, in this study, species classified as part of the genus Treponema include *berlinense*, *bryantii*, *succinifaciens*, *suis* and *porcinum*. While data related to these species is limited, *Treponema porcinum*, *Treponema succinifaciens*, *Treponema berlinense* and *Treponema bryantii* are associated with improved feed efficiency in pigs [20,21]. Further analysis such as shotgun metagenomics will need to be conducted to better understand the impact of changes in this genus have on the function of the microbiome. However, based on the other positive effects on gut health in pigs fed the LAM supplemented diet and the lack of negative impacts on gut health, it seems the changes in the Spirochaetes phylum are not negatively impacting the pigs.

Pigs fed both the LAM and the FUC supplemented diets had reduced bacterial diversity based on the Observed, Chao1, ACE and Fisher measures of alpha diversity. While there are conflicting opinions in the literature, lower bacterial diversity is generally considered to have negative connotations as greater microbial diversity is directly associated with ecosystem stability [22]. For example, in humans, reductions in diversity is associated with a range of health issues and diseases [23]. Therefore, while a reduced diversity in the pigs fed the FUC supplemented agrees with the changes in the microbiome and performance for this group, the reduction in diversity in pigs fed the LAM supplemented diet is surprising. The changes in the microbiome in this group particularly in the reduction in *Campylobacter* suggest improved gut health and therefore, it is unlikely that a reduction in diversity is having a negative influence in this group of pigs.

In relation to the gene expression data in this study, there are contrasting effects between the diets supplemented with the seaweed extracts. In relation to supplementation with LAM only minor changes in gene expression were identified relative to the control while more substantial changes were identified in pigs fed the FUC supplemented diet relative to pigs fed the control diet. However, there was region specific differences. In the duodenum, pigs fed the FUC supplemented diet had increased expression of SLC6A19 and CNDP1 compared to pigs fed the basal and also the pigs fed the diet supplemented with LAM. The two SWE supplemented groups had reduced expression of SLC16A10 compared to pigs fed the basal non-supplemented diet. These three transporters are all involved in the transport of amino acids [24]. This suggests there is increased availability and transport of amino acids in the duodenum of pigs fed the diets containing seaweed extracts compared to pigs fed the control. However, nutrient transporters are regulated by dietary substrate levels meaning the increased expression of protein transporters is a response to an increase in nutrient availability in the duodenum which can be impacted by a number of factors such as feed intake, but also may be a response to changes in intestinal architecture such as reductions in villus height [25]. A further influence of the SWE on nutrient transport was identified in the jejunum with pigs fed the FUC supplemented diets having increased expression of MCT1/SLC16A. SLC16A1 is responsible for the transport of monocarboxylates such as L-lactate and ketone bodies and also with the transport of butyrate [26,27]. These data, while speculative suggest an increased bacterial fermentation in the jejunum with the addition of FUC leading to increased production of butyrate and subsequent absorption with MCT1. While the influence of bacterial fermentation in small intestine is not as pronounced as the large intestine, the production and utilisation of butyrate in the small intestine and in particular the jejunum, as is the case in this study is important in the modulation of balance between apoptosis and proliferation [28]. In contrast to the upregulation of expression of genes related to nutrient digestion and absorption in the duodenum and jejunum, in the ileum, FUC supplementation led to a reduction in the expression of SLC2A2, GCG, FABP2, SGLT1 and SI in the ileum. Similar, results were identified where the feeding of a similar level of FUC in the post-weaning period had a similar influence on nutrient transporters suggesting that the addition of FUC has an impact in nutrient availability [6]. In the study of Rattigan et al. [6] the authors attributed the reduction in gene expression to the presence of alginates and fucoidan in the extract. Both FUC and alginates have been attributed with increasing the viscosity of digestive contents, reducing the flow of digesta and reducing the mixing of digestive contents leading to lower rates of nutrient breakdown [29,30]. The impact of these contrasting effects in different regions of the small intestine are difficult to quantify particularly due to the fact ileal digestibility’s was not measured. Unfortunately, the material required to conduct an analysis of nutrient digestibility was unavailable due to the volume of digesta in the digestive tract at the time of slaughter. Further research will be required to further understand the impacts of these extracts and nutrient breakdown and absorption.

Significant effects were identified between experimental groups in both the expression of targets related to barrier function and the expression of cytokines. Pigs fed the FUC supplemented diet had reductions in the expression of *OCLN* in both the jejunum and ileum compared to pigs fed the basal diet, while a tendency towards reduced expression was also identified in the duodenum. In agreement with these data is a reduction in the expression of *CLDN3* and *CLDN5* in the ileum compared to pigs fed the control diet. Proteins in the claudin and occludin families are a main component of tight junctions and form a seal that modulates paracellular transport in the intestinal epithelium [31]. Reduced expression of claudins such as 3 and 5 is associated with intestinal inflammatory disorders [32]. This is in agreement with effect identified on the expression of IL8/CXCL8 in the jejunum and the increased expression of *TGFB1* and *TNF* in the ileum of pigs fed the diet supplemented with FUC compared to pigs fed the basal unsupplemented diet. For the pigs fed the FUC supplemented diet the changes in gene expression are likely indicators of detrimental effects of the extract on gut health with increased markers of inflammation and reduced gene expression of targets associated with barrier function. In conjunction with the changes in the microbiome in the large intestine these data are possible explanations for the reduction in performance of pigs fed FUC. The pigs fed LAM had reductions in the expression of *MUC1* compared to pigs fed the basal and also pigs fed the diet supplanted with LAM. MUC1 provides a barrier against potential pathogens while also modulating the expression of inflammatory cytokines [33]. Therefore, the increase in expression of this gene is surprising as pigs fed the diet supplemented with LAM had no increases in inflammation, a healthier microbial profile and improved performance. Further research will need to be conducted to establish the reasons for this increase in expression such as a quantification of protein abundance as changes in expression are not always a true reflection of the production of the associated protein.

The aim of this study was to determine if the continued supplementation of two SWEs past the initial post-weaning period continues to be beneficial to performance. It is interesting to note that while SWE supplementation past the immediate post-weaning period up to d 35 in this study did not result in improved performance in healthy pigs under good sanitary experimental conditions, there was evidence to suggest that LAM supplementation had positive effects on gut health. This is significant as previous studies have identified that SWE supplementation can provide substantial performance and health benefits when the pigs become challenged. Heim at al [34] identified that SWE supplemented pigs had lower faecal scores and better performance than basal fed pigs following an ETEC challenge. Rattigan et al. [6] identified that LAM supplementation improves performance when pigs are housed in unsanitary conditions. While the mode of action is not completely understood, the evidence would suggest that bioactives in the SWE can influence both the host immune cells and gut microbiome cells. For instance, bioactives such as laminarin, can be internalized by intestinal epithelial cells and gut associated lymphoid cells [35]. This internalization upregulates the expression of pattern recognition receptors, increasing protective cytokine expression and induces protection against infectious challenge [36]. Hence, this effect would not be evident until the pigs undergo some type of environmental or pathogenic challenge.

## 4. Materials and Methods

All experimental procedures described in this work were approved under the University College Dublin Animal Research Ethics Committee (AREC-17-19-O’Doherty, 11 May, 2017) and were conducted in accordance with Irish legislation (SI no. 543/2012) and the EU directive 2010/63/EU for animal experimentation.

### 4.1. Experimental Design and Animal Management

72 weaned pigs (progeny of Landrace boars × (Large White × Landrace) sows) were sourced from sows from the same farrowing house. Blocking of piglets was done on the basis of initial live weight (8.4 kg, sd 1.05 kg), sex, litter of birth and pigs were assigned to one of three groups: 1) basal diet; 2) basal diet + 250ppm fucoidan rich extract (FUC); 3) basal diet + 300 ppm of a laminarin (LAM) rich extract with a total of 8 replicates per treatment.

The pigs were penned in groups of three and housed on fully slatted floors (1.68 × 1.22 m) with the initial temperature set at 30 °C and subsequently reduced each week by 2 °C with the humidity maintained at 65%. Pig weights were recorded on day 0 (weaning) and subsequently on days 7, 14, 21, 28 and 35. Feed and water were available ad libitum from four-space feeders and nipple drinkers, respectively, throughout the experiment.

### 4.2. Preperation of Experimental Diets and Characterisation of Extracts

Pigs in this experiment were offered one of three diets: (1) basal diet; (2) basal diet + 250 ppm FUC; (3) basal diet + 300 ppm LAM. The basal diet contained 15.3 MJ/kg digestible energy, 190 g/kg crude protein and 13.5 g/kg total lysine. The basal diet was comprised of wheat (34%), extruded full fat soya (17%), flaked wheat (13%) soya bean meal (48% crude protein) (10.5%), flaked maize (7%) whey powder (5%), provisoy (soya protein concentrate) (6.5%), soya oil (3%) with the remainder compromised of mineral and vitamin supplements (Table 7). Lysine was used a reference for calculation of the other amino acid requirements (NRC, 2012). The extraction procedures and methodology were described in previous publications but are further outlined below [5,6,37].

A laminarin-rich extract (BioAtlantis Ltd., Clash Industrial Estate, Tralee, Ireland) was obtained from *Laminaria Digitata* using a hydrothermal-assisted extraction and pre-optimized conditions for maximum yield of laminarin as described previously [38,39]. Briefly, dried and milled seaweed was suspended of 0.1N HCl maintaining a solid to liquid ratio of 1:21 (g/mL). The mixture was thoroughly agitated to ensure uniformity and then subjected to a temperature of 100 °C for 30 min. The subsequent crude extract was partially purified to increase the relative polysaccharide content and to remove or reduce other constituents; proteins, polyphenols, mannitol and alginate. This was achieved through mixing the crude extract with pure ethanol (to remove polyphenols) followed by water (to remove protein) and calcium chloride (to remove alginates).

A fucoidan-rich extract (BioAtlantis Ltd., Clash Industrial Estate, Tralee, Ireland) was obtained from *Ascophyllum nodosum* using a hydrothermal-assisted extraction and pre-optimized conditions for maximum yield of fucoidan [39]. The crude extract was partially purified to increase the relative polysaccharide content and to remove or reduce other constituents such as proteins, polyphenols, mannitol and alginate. This was achieved through mixing the crude extract with pure ethanol (to remove polyphenols) followed by water (to remove protein) and calcium chloride (to remove alginates). Fractions were separated based on molecular weight by employing a molecular weight cut-off centrifugal concentrator (100 KDa cut-off).

### 4.3. Post Slaughter Sample Collection

On d35 following slaughter, tissue sections of 1cm^2^ were cut from the duodenum, jejunum and ileum and washed using sterile PBS (Oxoid). The overlying smooth muscle was removed before storage in 5 mL RNAlater solution (Applied Biosystems, Dublin, Ireland) overnight at 4 °C which was subsequently removed before long term storage at –80 °C. Colonic digesta was removed and stored in sterile containers (Sarstedt, Wexford, Ireland) and frozen (–20 °C) for subsequent 16s rRNA sequencing and VFA analyses.

### 4.4. Volatile Fatty Acid Analysis

The VFA concentrations in the digesta were determined using gas liquid chromatography (GLC) as reported by Clarke et al. [41]. 1 g of digesta was mixed with distilled water (2.5 × sample weight) and centrifuged at 1400 g for 10 min (Sorvall GLC-2B laboratory centrifuge, DuPont, Wilmington, DE, USA). One mL of the subsequent supernatant and 1 mL of internal standard (0.05% 3-methyl-n-valeric acid in 0.15 M oxalic acid dihydrate) were mixed with 3 mL of distilled water. The reaction mixture was centrifuged at 500 g for 10 min, and the supernatant was filtered through 0.45 PTFE syringe filter into a chromatographic sample vial. An injection volume of 1 μL was injected into a Varian 3800 GC equipped with a EC™ 1000 Grace column (15 m × 0.53 mm I.D) with 1.20 μm film thickness. The temperature was set at 75–95 °C increasing by 3 °C/min, 95–200 increasing by 20 °C per minute, which was held for 0.50 min. The detector and injector temperature were 280 °C and 240 °C, respectively, while the total analysis time was 12.42 min.

### 4.5. Microbiological Analyses

#### 4.5.1. Microbial DNA Extraction

The extraction of microbial genomic DNA was conducted using a QIAamp DNA stool kit (Qiagen, West Sussex, UK) according to standard protocols. After DNA extraction DNA was evaluated using the Nanodrop spectrophotometer (Thermo Scientific, Wilmington, DE, USA).

#### 4.5.2. Illumina Sequencing

The V3–V4 hypervariable region of the bacterial 16S rRNA gene was performed on an Illumina MiSeq platform according to their standard protocols (Eurofins, Wolverhampton, UK). The V3–V4 region was PCR-amplified with universal primers taking in adapters including nucleotide sequences for forward and reverse index primers. Amplicon purification was conducted with AMPure XP beads (Beckman Coulter, Indianapolis, IN, USA) and prepared for index PCR using Nextera XT index primers (Illumina, San Diego, CA, USA). The purification step was repeated on the indexed samples using AMPure XP beads and assessed using a fragment analyzer (Agilent, Santa Clara, CA, USA). Following this step a pool was made using equal quantities from each experimental sample. The library was then analysed using the Bioanalyzer 7500 DNA kit (Agilent) and sequenced using the V3–V4 chemistry (2 × 300 bp paired-end reads).

### 4.6. Bioinformatic and Statistical Analyses

Quantitative Insights into Microbial Ecology (Qiime) was used to examine the sequencing data [42]. Sequencing primers were removed using the cutadapt package and the resulting paired end reads were merged using the paired-end reads function within Qiime using the standard criteria. Demultiplexing of paired end raw reads was through the split libraries function and quality filtering was conducted utilizing default QIIME parameters. Only reads that contained no ambiguous characters, no non-exact barcode matches, a sequence length > 225 nucleotides and a read-quality score of > 27 were retained. The uclust function in Qiime was used to pick OTUs based on a sequence similarity of 97%. Singletons were removed, as only OTUs that were present at the level of at least two reads in more than one sample were retained while chimeric sequences were removed using ChimeraSlayer [43,44]. The GreenGenes database assigned OTUs to different taxonomic levels. A combination of the mormalized OTU table, experimental phenotypic data and the phylogenetic tree were combined to produce the phyloseq object for further analysis (http://www.r-project.org; version 3.5.0, accessed on 25 March). This phyloseq R package was used to examine measures of richness and diversity using the Observed, Chao1, ACE, Shannon, Fisher and Simpson measures as described by Maurer et al. [45]. The PROC GLIMMIX procedure of Statistical Analysis Software (SAS) 9.4 (SAS Institute, Cary, NC, USA) was used to examine differences between experimental treatments at phylum, family and genus level with pig being the experimental unit and *P*-values presented using a Benjamini–Hochberg (BH) correction.

### 4.7. Nanostring nCounter Analysis

Tissue from the duodenum, jejunum and ileum were used for analysis of gene expression profiles using the Nanostring nCounter Analysis System (Nanostring Technologies, Seattle, Washington, USA). Gene lists contained six positive and eight negative controls, eight reference targets and thirty-two target genes. A single multiplexed hybridisation reaction, as originally described by Geiss et al. [46] was used to analyse all target genes. Samples were initially standardized to 20 ng/μL using a Qubit fluorometer (Thermo Fisher Scientific). A master mix was created by combining 70 μL of hybridisation buffer and the reporter codeset, as per the manufacturer instructions. For the hybridization reaction each tube contained the master mix (8 μL), the sample (5 μL) (total RNA concentration 100 ng) and capture probeset (2 μL). Each reaction tube was inverted to mix and spun down before incubation at 65 °C for 20 h in a Bio-rad thermocycler.

The Nanostring nCounter prep station liquid handling robot which was used for post-hybridisation processing. This involved the removal of excess unbound probes and immobilisation of samples onto the internal surface of the sample cartridge to allow imaging to be conducted using the digital analyser, which collects data by taking images of the immobilised fluorescent reporters in the sample cartridge with a CCD camera through a microscope objective lens.

The analysis and normalisation of the raw Nanostring data were performed using the nSolver Analysis Software v4.0 (Nanostring Technologies) which was used for initial data normalisation and analysis. The background threshold value was estimated using the average count of the negative control probes in every reaction plus 2 standard deviations to which all samples were adjusted [47]. Gene targets with raw counts below the threshold in more than two thirds of samples were excluded from the analysis. Raw counts were normalised using a combination of positive control normalisation and codeset content normalisation. The former accounts for errors such as pipetting errors, lot-to-lot variation in nCounter preparation plates and nCounter cartridges, while the latter uses reference/housekeeping genes to account for variability in the quantity and quality of sample RNA.

### 4.8. Analysis of Performance and Gene Expression Data

The univariate procedure of SAS 9.4 (SAS Institute, Cary, NC, USA) was used to check performance, gene expression and VFA data for normality. Experimental data were analysed as a complete randomized design using the mixed procedure of SAS with the fixed effect of treatment. The initial weight was used as a covariate for the performance data with pen being the experimental unit. For all other data, the pig was the experimental unit. The probability level that denoted significance was *P* < 0.05, while *P*-values between 0.05 and 0.1 are considered numerical tendencies. Data are presented as least-square means with their standard errors of the mean.

## 5. Conclusions

In conclusion, while it was hypothesized that both LAM and FUC extracts would have a beneficial impact on performance and intestinal health out to day 35 post-weaning, only the supplementation of LAM was beneficial on intestinal health, with no identifiable effect on performance. Further research will need to be conducted to assess the long-term impact of the feeding the LAM extract in unsanitary/challenge conditions, but also for inclusion in human studies due to its positive impact on the intestinal microbiome.

## Figures and Tables

**Table 1:** Biochemical composition of the extracts obtained following extraction and purification.

Component	LAM	FUC
Laminarin	653.2	25.9
Fucoidan	190	441
Phlorotannin	5	135
Mannitol	51	43.8
Alginates	40	34.8
(g/kg dry weight extract)		

**Table 2:** The effect of supplementation with either laminarin or fucoidan on animal performance.

	Basal	FUC250	Lam300	SEM	*P* Value
Weight d0	8.42	8.38	8.36	0.10	0.877
Weight d14	10.95	11.17	11.19	0.49	0.931
Weight d35	23.46	21.72	22.60	0.96	0.454
ADFI *	0.91	0.94	0.88	0.04	0.537
ADG	0.60	0.50	0.54	0.03	0.071
G:F	0.67 ^a^	0.54 ^b^	0.63 ^ab^	0.04	0.045

*ADFI: average daily feed intake; ADG: average daily gain; G:F: gain to feed ratio; ^a,b,^ values with different superscripts differ significantly (*P* < 0.05); A total of seven replicates were used per treatment group (replicate = pen, 3/pigs/pen).

**Table 3:** The effect of supplementation with either laminarin or fucoidan on volatile fatty acids (VFAs) produced in colonic digesta on d35 post-weaning.

	Basal	FUC250	Lam300	SEM	*P* Value
VFA (mmol/l digesta)					
Total	154.76 ^ab^	149.89 ^a^	172.45 ^b^	9.19	0.041
Acetate	119.75	109.64	127.43	7.80	0.214
Propionate	26.20	26.87	27.68	2.82	0.919
Butyrate	7.36 ^a^	11.83 ^b^	15.52 ^c^	1.54	0.003
Valerate	0.77	0.83	0.93	0.13	0.724
Isobutyrate	0.30	0.34	0.41	0.05	0.356
Isovalerate	0.38	0.39	0.50	0.08	0.479
Ace:Pro	4.97	4.26	4.95	0.61	0.666
Protein Derived SCFA	1.46	1.56	1.83	0.22	0.481

Ace:Pro: Acetate to propionate ratio, Seven replicates per treatment used; ^a,b,c,^ values with different superscripts differ significantly (*P* < 0.05); A total of seven replicates were used per treatment group (replicate = pig).

**Table 4:** Effects of laminarin and fucoidan supplementation on measures of alpha diversity in colonic digesta.

	Basal	FUC	LAM300	SEM	*P* Value
Observed	179.00 ^a^	158.71 ^b^	158.14 ^b^	4.51	0.016
Chao1	179.94 ^a^	159.84 ^b^	158.70 ^b^	4.55	0.018
ACE	180.06 ^a^	159.88 ^b^	158.99 ^b^	4.55	0.018
Shannon	4.49 ^a^	4.07 ^b^	4.29 ^b^	0.05	0.047
Fisher	39.61 ^a^	33.96 ^b^	33.84 ^b^	1.17	0.016
Simpson	0.98	0.96	0.97	0.01	0.095

^a,b,^ values with different superscripts differ significantly (*P* < 0.05); A total of seven replicates were used per treatment group (replicate = pig).

**Table 5:** Assessment of microbial composition in colonic digesta.

	Basal *	FUC250	LAM300	SEM	Adj*P*
**Phylum**					
Bacteroidetes	65.76	55.59	60.23	3.01	0.140
Firmicutes	20.29 ^a^	18.14 ^a^	27.98 ^b^	1.81	0.005
Proteobacteria	11.08 ^a^	22.47 ^b^	3.39 ^c^	1.32	0.001
Spirochaetes	1.21 ^a^	2.48 ^a^	5.70 ^b^	0.61	0.001
**Family**					
*Ruminococcaceae*	8.61 ^a^	5.11 ^b^	10.65 ^a^	1.07	0.011
*Campylobacteraceae*	4.74 ^a^	17.67 ^b^	0.21 ^c^	0.97	0.001
*Spirochaetaceae*	1.23 ^a^	2.47 ^a^	5.91 ^b^	0.61	0.003
**Genus**					
*Faecalibacterium*	7.11 ^a^	3.03 ^b^	8.39 ^a^	0.94	0.005
*Alloprevotella*	6.78 ^a^	2.17 ^b^	1.72 ^b^	0.79	0.001
*Campylobacter*	4.75 ^a^	17.72 ^b^	0.21 ^c^	0.97	0.001
*Roseburia*	4.26 ^a^	6.01 ^ab^	7.52 ^b^	0.98	0.007
*Treponema*	1.23 ^a^	2.47 ^a^	5.89 ^b^	0.61	0.001

^a,b,c,^ values with different superscripts differ significantly (*P* < 0.05); * A total of seven replicates were used per treatment group (replicate = pig).

**Table 6:** Effect of laminarin or fucoidan inclusion on the gene expression of nutrient transporters and immune markers in the duodenum, jejunum and ileum.

	Basal	FUC250	Lam300	SEM	*P* Value
**Nutrient transporters and digestive enzymes**					
Duodenum					
*SLC6A19*	2557.15 ^ab^	2839.39 ^b^	1954.65 ^a^	217.85	0.030
*CNDP1*	320.60 ^a^	472.57 ^b^	301.63 ^a^	48.77	0.045
*SLC16A10*	568.63 ^a^	418.27 ^b^	443.36 ^b^	41.29	0.042
Jejunum					
*SLC16A1*	1182.78 ^a^	1587.81 ^b^	1230.59 ^a^	79.36	0.004
Ileum					
*SLC2A2*	2108.30 ^a^	436.09 ^b^	1438.96 ^ab^	420.96	0.038
*GCG*	4530.86 ^a^	1351.68 ^b^	2844.24 ^ab^	812.76	0.041
*FABP2*	21949.73 ^a^	4078.26 ^b^	12809.36 ^ab^	4594.29	0.042
*SLC5A1*	16134.62 ^a^	3930.36 ^b^	9226.85 ^ab^	3156.59	0.042
*SI*	46058.26 ^a^	12573.66 ^b^	25949.67 ^ab^	8748.13	0.043
*CNDP1*	310.73 ^a^	161.77 ^b^	160.23 ^b^	46.13	0.048
*OCLN*	2464.03 ^a^	786.38 ^b^	1782.07 ^ab^	442.92	0.048
**Markers of immune response and intestinal integrity**					
Jejunum					
*CXCL8*	1042.45 ^a^	1815.05 ^b^	1121.75 ^a^	194.7	0.022
*MUC1*	41.70 ^a^	45.81 ^a^	26.89 ^b^	3.61	0.004
*OCLN*	5209.83 ^a^	3788.43^b^	4300.55 ^ab^	329.86	0.022
Ileum					
*TGFB1*	240.40 ^a^	325.40 ^b^	235.21 ^a^	23.64	0.029
*TNF*	79.88 ^a^	136.30 ^b^	85.51 ^a^	15.26	0.037
*CLDN5*	115.14 ^a^	66.00 ^b^	83.2 ^ab^	13.14	0.046
*CLDN3*	2281.14 ^a^	694.20 ^b^	1501.2 ^ab^	416.79	0.047
*OCLN*	2464.03 ^a^	786.38 ^b^	1782.07 ^ab^	442.92	0.048

^a,b,^ values with different superscripts differ significantly (*P* < 0.05); A total of seven replicates were used per treatment group (replicate = pig).

**Table 7:** Ingredient and chemical composition of basal diet *.

Ingredient (g/kg)	
Wheat	380.0
Barley	233.0
Soya bean meal	170.0
Full fat soya	120.0
Whey powder	50.0
Dicalcium phosphate	13.0
Soya oil	10.0
Calcium carbonate (Limestone)	11.0
Lysine HCL	4.0
Vitamins and minerals	3.0
Salt	3.0
DL-methionine	1.5
l-threonine	1.5
**Chemical analysis**	
DM	866.1
Crude protein (N × 6.25)	190
Digestible energy (MJ/kg) ^†^	14.95
Ash	48.4
Neutral detergent fibre	114.00
Lysine ^†^	13.5
Methionine and cysteine ^†^	7.4
Threonine ^†^	7.9
Tryptophan ^†^	2.6
Calcium ^†^	7.2
Phosphorous ^†^	6.0

* Treatments were as follows: (1) basal diet; (2) basal diet; basal diet + 250 parts per million (ppm) fucoidan; (3) basal diet + 300 ppm of a laminarin rich extract basal diet; ^†^ Values calculated based on tabulated nutritional composition [40]. The basal diet was formulated to provide: Cu, 100; Fe, 140; Mn, 47; Zn, 120; I, 0.6; Se, 0.3; retinol, 1.8; cholecalciferol, 0.025; α-tocopherol, 67; phytylmenaquinone, 4; cyanocobalamin, 0.01; riboflavin, 2; nicotinic acid, 12; pantothenic acid, 10; choline chloride, 250; thiamine, 2; pyridoxine, 0.015 (mg/kg diet). Celite was also included at 300 mg/kg.

## Supplementary Materials

The following are available online at https://www.mdpi.com/article/10.3390/md19040183/s1, Table S1: Differential abundance analysis of bacterial taxa at phylum level in colonic digesta, Table S2: Differential abundance analysis of bacterial taxa at family level in colonic digesta, Table S3: Differential abundance analysis of bacterial taxa at genus level in colonic digesta, Table S4: Effect of laminarin or fucoidan inclusion on the expression of nutrient transporters and immune markers in the duodenum, Table S5: Effect of laminarin or fucoidan inclusion on the expression of nutrient transporters and immune markers in the jejunum, Table S6: Effect of laminarin or fucoidan inclusion on the expression of nutrient transporters and immune markers in the ileum.
