# Supplementary material for: Effect of Supplementing Seaweed Extracts to Pigs until d35 Post-Weaning on Performance and Aspects of Intestinal Health
Source: Mar Drugs. 2021 Mar 26;19(4):183. doi: 10.3390/md19040183 (PMC8066862; doi:10.3390/md19040183)
Supplement: Supplementary file 1 [file marinedrugs-19-00183-s001.pdf]

Table S1: Differential abundance analysis of bacterial taxa at phylum level in colonic digesta

	**Basal**	**FUC250**	**LAM300**	**SEM**	**AdjP**
Bacteroidetes	65.76	55.59	60.23	3.00	0.140
Firmicutes	20.29[ab]	18.14[a]	27.98[b]	1.81	**0.005**
Proteobacteria	11.08[a]	22.47[b]	3.39[c]	1.32	**0.001**
Spirochaetes	1.21[a]	2.48[a]	5.70[b]	0.61	**0.000**
Actinobacteria	0.57	0.32	0.38	0.23	0.719
Tenericutes	0.51	0.25	0.75	0.25	0.537
Candidatus Melainabacteria	0.17	0.24	0.51	0.17	0.512

[*] Six replicates per treatment used

Table S2: Differential abundance analysis of bacterial taxa at family level in colonic digesta

	Basal	FUC250	LAM300	SEM	AdjP
Prevotellaceae	64.99	55.24	59.67	2.98	0.167
Ruminococcaceae	8.61[a]	5.11[b]	10.65[a]	1.07	**0.011**
Lachnospiraceae	7.64	8.96	11.70	1.20	**0.101**
Campylobacteraceae	4.74[a]	17.67[b]	0.21[d]	0.97	**0.000**
Burkholderiaceae	2.59	1.13	1.37	0.49	0.194
Spirochaetaceae	1.23[a]	2.47[a]	5.91[b]	0.61	**0.003**
Succinivibrionaceae	1.12	0.35	0.30	0.26	0.164
Pasteurellaceae	0.78	1.59	0.49	0.39	0.190
Oscillospiraceae	0.77	0.24	0.53	0.28	0.623
Selenomonadaceae	0.74	0.65	1.33	0.35	0.482
Rikenellaceae	0.67	0.91	1.01	0.35	0.901
Muribaculaceae	0.60	0.13	0.66	0.25	0.518
Propionibacteriaceae	0.48	0.16	0.29	0.19	0.642
Clostridiaceae	0.43	0.40	0.41	0.24	0.998
Sutterellaceae	0.41	0.46	0.43	0.24	0.965
Mycoplasmataceae	0.40	0.21	0.65	0.22	0.522
Rhodospirillaceae	0.32	0.05	0.32	0.16	0.653
Hungateiclostridiaceae	0.29	0.47	0.64	0.23	0.537
Veillonellaceae	0.17	0.19	0.18	0.16	0.995
Acetobacteraceae	0.14	0.89	0.23	0.18	0.161
Anaeroplasmataceae	0.12	0.04	0.13	0.12	0.957
Bacteroidaceae	0.11	0.03	0.03	0.09	0.919
Helicobacteraceae	0.08	0.19	0.12	0.13	0.915
Christensenellaceae	0.06	0.06	0.05	0.08	0.994
Streptococcaceae	0.05	0.24	0.01	0.12	0.710
Lactobacillaceae	0.03	0.19	0.06	0.17	0.163
Erysipelotrichaceae	0.03	0.02	0.16	0.10	0.822
Acidaminococcaceae	0.01	0.05	0.03	0.07	0.983

[*] Six replicates per treatment used

Table S3: Differential abundance analysis of bacterial taxa at genus level in colonic digesta

Genus	Basal	FUC250	LAM300	SEM	AdjP
Prevotella	49.78	46.09	49.13	2.66	0.674
Prevotellamassilia	8.53	6.88	8.41	1.06	0.421
Faecalibacterium	7.11[a]	3.03[b]	8.39[a]	0.94	**0.005**
Alloprevotella	6.78[a]	2.17[b]	1.72[b]	0.79	**0.001**
Campylobacter	4.75[a]	17.72[b]	0.21[c]	0.97	**0.000**
Roseburia	4.26[a]	6.01	7.52[b]	0 .98	**0.007**
Ralstonia	2.17	0.83	1.36	0.46	0.265
Lachnoclostridium	1.28	0.72	0.66	0.36	0.624
Treponema	1.23[a]	2.47[ab]	5.89[ab]	0.61	**0.001**
Succinivibrio	1.09	0.32	0.3	0.26	0.165
Gemmiger	0.98	1.05	0.71	0.35	0.845
Anaerovibrio	0.74	0.65	1.33	0.35	0.488
Oscillibacter	0.73	0.24	0.52	0.28	0.653
Eubacterium	0.71	0.82	0.88	0.33	0.885
Alistipes	0.67	0.91	1.01	0.34	0.785
Glaesserella	0.64	1.18	0.4	0.32	0.391
Oribacterium	0.6	0.25	0.22	0.22	0.631
Muribaculum	0.6	0.13	0.66	0.25	0.522
Pseudobutyrivibrio	0.54	0.55	1.25	0.3	0.234
Propionibacterium	0.48	0.16	0.28	0.19	0.645
Mycoplasma	0.39	0.21	0.64	0.22	0.530
Clostridium	0.39	0.37	0.3	0.23	0.994
Sutterella	0.35	0.36	0.3	0.24	0.890
Kineothrix	0.29	0.39	0.38	0.21	0.924
Ruminococcus	0.19	0.35	0.52	0.21	0.726
Dialister	0.18	0.19	0.18	0.16	0.995
Vampirovibrio	0.17	0.24	0.53	0.17	0.498
Ruminiclostridium	0.16	0.16	0.32	0.15	0.771
Acetobacter	0.14	0.89	0.23	0.18	0.162
Actinobacillus	0.13	0.34	0.07	0.21	0.135
Anaerobacterium	0.13	0.31	0.32	0.17	0.713
Anaeroplasma	0.12	0.04	0.13	0.12	0.958
Helicobacter	0.08	0.19	0.12	0.13	0.917
Anaerostipes	0.06	0.14	0.48	0.14	0.356
Christensenella	0.06	0.06	0.05	0.08	0.994
Duodenibacillus	0.05	0.09	0.13	0.12	0.958
Murimonas	0.05	0.04	0.09	0.1	0.969
Streptococcus	0.05	0.24	0.01	0.12	0.713
Butyricicoccus	0.04	0.03	0.11	0.09	0.927
Lactobacillus	0.03	0.19	0.06	0.17	0.665

Turicibacter	0.03	0.02	0.16	0.1	0.821
Blautia	0.02	0.02	0.34	0.16	0.433
Phascolarctobacterium	0.01	0.02	0.01	0.05	0.988
Intestinimonas	0.01	0.06	0.1	0.08	0.910

Table S4: Effect of laminarin or fucoidan inclusion on the expression of nutrient transporters and immune markers in the duodenum

	Basal	FUC250	Lam300	SEM	P value
SLC6A19	2557.15[ab]	2839.39[b]	1954.65[a]	217.85	**0.030**
SLC16A10	568.63[a]	418.27[b]	443.36[b]	41.29	**0.042**
CNDP1	320.60[a]	472.57[b]	301.63[a]	48.77	**0.045**
PEPT1/SLC15A1	3787.42[ab]	4759.22[a]	3418.55[b]	379.13	0.059
AMY2	267.45[a]	214.06[b]	218.64[ab]	16.92	0.072
OCLN	2257.51[a]	1885.58[b]	1925.19[ab]	124.72	0.096
FABP2	40881.27[a]	33579.91	27469.65[b]	4122.65	0.098
SMCT1/SLC5A8	4118	3915.99	4639.94	279.18	0.195
GLUT2/SLC2A2	3476.31	3048.59	2391.42	430.92	0.228
MUC2	8089.42	11356.98	9517.93	1303.71	0.234
TNF-α	18.42	19.4	25.79	3.32	0.259
SLC7A1	144.31	165.65	156.94	9.31	0.289
GLUT7/SLC2A7	618.08	497.57	449.48	75.52	0.291
CLDN5	57.4	48.01	56.29	5.12	0.386
IL-1α	34.88	50.88	45.33	8.42	0.413
MCT1/SLC16A1	2780.13	2431.56	2718.27	208.52	0.467
ZO-1	973.92	863.48	897.08	70.96	0.541
GLP2R	187.18	163.2	172.43	15.96	0.573
SI	21297.55	18202.83	16861.08	3040.67	0.581
IL-8	1216.62	1551.94	1542.47	259.41	0.591
TLR5	114.59	96.67	119.76	17.17	0.616
SGLT1/SLC5A1	7985.91	7756.24	6442.97	1203.29	0.627
TGF-β	115.02	119.76	111.67	6.73	0.7
GHRL	77.47	72.71	97.41	22.17	0.71
CLDN3	2426.27	2284.91	2296.92	133.16	0.712
GLUT5	866.41	907.06	800.63	123.84	0.83
CCK	1166.42	1199.37	1313.59	183.92	0.84
NPY	31.31	30.38	35.03	6.01	0.847
IL-1β	21.95	26.33	27.24	7.39	0.865
TLR2	96.49	106.23	93.76	17.18	0.866
GLUT1/SLC2A1	393.4	409.8	423.45	44.14	0.891
NFKB1	550.02	529.23	525.78	44.59	0.918
TLR4	139.31	129.3	134.61	26.48	0.965
IFNγ	84.16	85.55	87.32	8.55	0.966
MUC1	47.47	46.6	45.12	6.68	0.969
GCG	142.83	129.65	134.99	45.71	0.979

GLUT8	419.14	415.46	420.48	58.72	0.998

Table S5: Effect of laminarin or fucoidan inclusion on the expression of nutrient transporters and immune markers in the jejunum

	Basal	FUC250	LAM300	SEM	P value
MCT1/SLC16A1	1182.78^{a}	1587.81^{b}	1230.59^{b}	79.36	**0.004**
MUC1	41.70^{a}	45.81^{a}	26.89^{b}	3.61	**0.004**
IL-8	1042.45^{a}	1815.05^{b}	1121.75^{a}	194.7	**0.022**
OCLN	5209.83^{a}	3788.43^{b}	4300.55a^{b}	329.86	**0.022**
FABP2	129816.69^{a}	66943.58^{b}	110219	17678.58	0.06
TLR5	344.47^{a}	241.94^{b}	301.27	28.85	0.066
GLUT7/SLC2A7	879.30^{a}	517.42^{b}	755.64	109.37	0.086
CLDN5	170.92^{a}	135.62	125.35^{b}	15.11	0.11
SLC16A10	1576.06^{a}	1029.45^{b}	1142.51	189.22	0.126
GLP2R	256.73^{a}	195.61^{b}	228.18	20.58	0.139
SI	81368.00^{a}	58990.03^{b}	70420.17	7621.34	0.145
IL-1α	55.17^{a}	82.91^{b}	60.07	10.3	0.156
IFNγ	312.59	348.64	245.18	37.02	0.163
CNDP1	547.57^{a}	419.01^{b}	494.19	49.2	0.207
CLDN3	3622.96	2992.04	3031.69	281.5	0.234
AMY2	427.62	343.22	342.98	39.73	0.248
SLC6A19	5528.2	4282.34	4837.1	511.06	0.252
TLR2	550.34	685.95	469.54	90.44	0.258
GLUT1/SLC2A1	311.35	438.09	337.08	60.7	0.319
GLUT2/SLC2A2	5630.45	4304.08	5016.24	605.47	0.324
SGLT1/SLC5A1	25745.23	19135.58	24049.21	3281.53	0.356
NPY	53.12	60.89	43.02	8.64	0.362
IL6	25.98	31.39	33.46	3.9	0.394
MUC2	11427.43	13524.25	10300.55	1662.67	0.399
IL-1β	41.61	62	48.77	10.79	0.417
TASR1	33.67	29.25	36.61	4.4	0.504
SMCT1/SLC5A8	5217.69	4755.08	4517.95	437.58	0.528
TGF-β	207.46	216.21	193.07	14.87	0.551
GLUT5	1141.33	885.33	1024.14	172.92	0.587
CCK	227.04	191.83	222.58	29.49	0.662
ZO-1	1931.78	1806.35	1785.86	124.78	0.676
PEPT1/SLC15A1	6798.21	6700.89	7430.39	678.73	0.716
GCG	4754.93	5240.79	4694.9	543.25	0.742
TLR4	205.12	237.64	206.86	35.04	0.765
TNF-α	48.48	50.58	55.16	6.67	0.772
SLC7A1	233.8	240.09	266.31	36.8	0.805
NFKB1	1043.16	1037.98	991.67	68.85	0.845
GLUT8	56.88	55.32	54.22	4.55	0.918

Table S6: Effect of laminarin or fucoidan inclusion on the expression of nutrient transporters and immune markers in the ileum

	Basal	FUC250	Lam300	SEM	P value
TGF-β	240.40[a]	325.40[b]	235.21[a]	23.64	**0.029**
TNF-α	79.88[a]	136.30[b]	85.51[a]	15.26	**0.037**
GLUT2/SLC2A2	2108.30[a]	436.09[b]	1438.96[ab]	420.96	**0.038**
GCG	4530.86[a]	1351.68[b]	2844.24[ab]	812.76	**0.041**
FABP2	21949.73[a]	4078.26[b]	12809.36[ab]	4594.29	**0.042**
SGLT1/SLC5A1	16134.62[a]	3930.36[b]	9226.85[ab]	3156.59	**0.042**
SI	46058.26[a]	12573.66[b]	25949.67[ab]	8748.13	**0.043**
CLDN5	115.14[a]	66.00[b]	83.2[ab]	13.14	**0.046**
CLDN3	2281.14[a]	694.20[b]	1501.2[ab]	416.79	**0.047**
CNDP1	310.73[a]	161.77[b]	160.23[b]	46.13	**0.048**
OCLN	2464.03[a]	786.38[b]	1782.07[ab]	442.92	**0.048**
SLC16A10	1246.36	551.67	713.85	208.19	0.068
TLR5	189.37	81.55	161.34	246.7	0.073
PEPT1/SLC15A1	2684.54	916.82	2156.51	523.75	0.077
AMY2	336.68	181.22	232.04	47.91	0.087
GLP2R	150.28	69.73	102.89	24.93	0.096
ZO-1	1696.71	1287.45	1404.82	131.21	0.098
SLC6A19	2755.6	1074.56	2010.45	519.82	0.099
MUC1	44.96	27.34	29.06	6.19	0.106
SLC7A1	330.60	511.02	376.88	59.14	0.113
SMCT1/SLC5A8	3623.60	1472.04	2724.05	689.83	0.114
MUC2	10126.43	5986.13	6897.12	1443.24	0.125
GLUT5	504.00	215.28	381.35	102.23	0.162
IL-8	881.37	525.81	682.58	138.4	0.212
GLUT7/SLC2A7	220	82.86	200	62.44	0.277
GLUT1/SLC2A1	276.08	334.29	295.69	25.23	0.278
IL-6	39.25	58.37	47.38	8.44	0.296
TLR2	952.17	700.13	681.8	135.15	0.296
IFNg	180.74	135.49	165.23	20.46	0.306
GLUT8	58	68.67	59.59	5.14	0.316
NFKB1	1084.05	1191.92	1061.71	63.37	0.338
TLR4	203.56	224.91	171.48	26.63	0.4
CCK	117.23	154.91	119.28	21.56	0.409
IL-1β	26.64	39.32	25.75	8.24	0.456
IL-1α	62.46	76.03	57.58	10.56	0.473

MCT1/SLC16A1	1406.88	1434.18	1371.95	85.21	0.881
NPY	52.43	59.01	56.25	10.53	0.904
MUC4	34.58	33.08	30.84	8.95	0.956
Tas1R1	37.44	38.64	38.94	5.94	0.982
